# Focal epilepsy disrupts spindle structure and function

**DOI:** 10.1038/s41598-022-15147-0

**Published:** 2022-07-01

**Authors:** Katharina Schiller, Tamir Avigdor, Chifaou Abdallah, Viviane Sziklas, Joelle Crane, Ambra Stefani, Laure Peter-Derex, Birgit Frauscher

**Affiliations:** 1 Analytical Neurophysiology Lab, Montreal Neurological Institute and Hospital, McGill University, 3801 University Street, Montreal, QC H3A 2B4 Canada; 2 Department of Pediatrics, Hospital Group Ostallgaeu-Kaufbeuren, Kaufbeuren, Germany; 3 Department of Neurology, Medical University Innsbruck, Innsbruck, Austria; 4 Center for Sleep Medicine and Respiratory Diseases, Croix-Rousse Hospital, University Hospital of Lyon, Lyon 1 University, Lyon, France; 5 Department of Medicine and Centre for Neuroscience Studies, Queen’s University, Kingston, ON Canada

**Keywords:** Epilepsy, Neurology

## Abstract

Sleep spindles are the hallmark of N2 sleep and are attributed a key role in cognition. Little is known about the impact of epilepsy on sleep oscillations underlying sleep-related functions. This study assessed changes in the global spindle rate in patients with epilepsy, analysed the distribution of spindles in relation to the epileptic focus, and performed correlations with neurocognitive function. Twenty-one patients with drug-resistant focal epilepsy (12 females; mean age 32.6 ± 10.7 years [mean ± SD]) and 12 healthy controls (3 females; 24.5 ± 3.3 years) underwent combined whole-night high-density electroencephalography and polysomnography. Global spindle rates during N2 were lower in epilepsy patients compared to controls (mean = 5.78/min ± 0.72 vs. 6.49/min ± 0.71, p = 0.02, d =  − 0.70). Within epilepsy patients, spindle rates were lower in the region of the epileptic focus compared to the contralateral region (median = 4.77/min [range 2.53–6.18] vs. 5.26/min [2.53–6.56], p = 0.02, rank biserial correlation RC =  − 0.57). This decrease was driven by fast spindles (12–16 Hz) (1.50/min [0.62–4.08] vs. 1.65/min [0.51–4.28], p = 0.002, RC =  − 0.76). The focal reduction in spindles was negatively correlated with two scales of attention (r =  − 0.54, p = 0.01; r =  − 0.51, p = 0.025). Patients with focal epilepsy show a reduction in global and local spindle rates dependent on the region of the epileptic focus. This may play a role in impaired cognitive functioning. Future work will show if the local reduction in spindles can be used as potential marker of the epileptic focus.

## Introduction

Sleep and epilepsy are linked in a bidirectional complex way^1^. On the one hand, disturbed sleep is known to worsen epilepsy, whereas, on the other hand, epilepsy has a negative impact on sleep^2^. Sleep macro- and micro-architecture were found to be disturbed in epilepsy patients, with non-rapid eye movement (NREM) sleep facilitating epileptic activity, and rapid eye movement (REM) sleep inhibiting epileptic activity^3,4^. Despite recent advances in the understanding of the relationship between epileptic activity and sleep microstructure^5^, neurophysiological quantitative studies pairing results with neurocognitive testing remain sparse.

Sleep spindles, characterized by a duration of > 0.5 s and a frequency of 10–16 Hz, are the hallmark of N2 NREM sleep and play an important role in memory and learning^6^. In particular, procedural and declarative memory, processing speed, and full-scale intelligence quotient have been positively correlated with spindle activity^6,7^. Fast spindles with a frequency of 12 to 16 Hz were specifically attributed to memory and processing speed in healthy individuals^7,8^. Several studies point to the fact that spindles might be altered in patients with epilepsy^9–11^. In children with primary generalized epilepsy, spindle reductions in N2 were reported on overnight recording with a lower frequency of fast spindles (12–16 Hz) at the end of the night compared to healthy controls^10^.

The extent of disturbances in spindle rates (number per minute) in individuals with focal epilepsy and patterns of fluctuation across the night remain poorly understood. Due to improved spatial coverage, high-density electroencephalogram (HD-EEG) allows for an excellent spatio-temporal resolution of cortical activity during sleep and epileptic activity. HD-EEG is therefore the ideal tool to explore the influence of the epileptic focus on spindles within the lobe of the focus and outside of the lobe of the focus. Until now, only one study has examined the relationship between spindles and neuropsychological performance. In this study, children with childhood epilepsy with centrotemporal spikes had reduced spindle rates in the centrotemporal regions during a nap and lower spindle rates were correlated to lower cognitive functioning^12^. However, this report is from a very specific, self-limited epilepsy syndrome, and findings cannot be generalized to other focal epilepsies and to the adult population. A comprehensive study of spindles in the adult epileptic brain, irrespective of the type of focal epilepsy and across a whole-night recording, which capitalizes on improved whole-brain coverage using HD-EEG is lacking. This question is of high clinical relevance as the demonstration of a local and/or remote alteration of these oscillations may contribute to the understanding of disturbed sleep architecture and cognitive dysfunction frequently encountered in people with epilepsy^13^.

In the current study, we use combined polysomnography and HD-EEG full-night sleep recordings in order to assess changes in the global spindle activity in adult patients with focal epilepsy compared to healthy controls. We decided to focus on analysing the rate of spindles which was shown to be altered in patients with epilepsy^10,12^ and spindle duration which may be influenced by epileptic activity and seizures^14^. Further, we analyse the distribution of spindles in relation to the epileptic focus and perform correlations with neurocognitive functions. More specifically, we aimed to (i) explore the global and regional extent of the alterations in spindle rates (ii) assess if these alterations were under sleep homeostatic control, (iii) analyse if epileptic activity assessed by spike rate influenced spindle rates, and (iv) investigate if alterations in spindle rates correlated with neurocognitive functions.

## Materials and methods

### Study population

From a total of 25 patients with unilateral drug-resistant epilepsy who underwent HD-EEG at the Montreal Neurological Institute and Hospital between January 2019 and May 2021, we selected 21 patients with focal epilepsy (12 females; mean age 32.6 ± 10.7 years [mean ± SD] (18–51 years)). Reasons for exclusion were previous brain surgery (n = 1), ≥ 10 electrodes with artifacts (n = 2), and interference of epileptic activity with sleep scoring (n = 1). Eleven of 21 patients (52.4%) had temporal lobe epilepsy; the remaining 10 patients had extratemporal lobe epilepsy. None of the patients had overall cognitive impairment (intelligence quotient below 70), including neurodevelopmental delay. The epileptic focus was defined based on presurgical evaluation consisting of long-term video-EEG monitoring, anatomical 3T magnetic resonance imaging, positron emission tomography and neuropsychological evaluation and results of invasive intracranial EEG, if available. The epileptic focus was categorized into superficial, deep or intermediate depending on its depth^15^. Demographic characteristics of the patients are reported in Table 1. Twelve healthy controls (3 females; 24.5 ± 3.3 years (20–29 years)) were recruited through advertisements posted at the Centre for Neuroscience Studies at Queen’s University. Healthy controls had no sleep disorders and no neurological diseases. The study protocol was approved by the Research Ethics Board at the Montreal Neurological Institute and Hospital (2022-8115). All study participants provided written informed consent in agreement with the Research Ethics Board at the Montreal Neurological Institute and Hospital and Queen’s University. All methods were performed in accordance with the relevant guidelines and regulations.

**Table 1:** Patient demographics.

ID	Age, Sex	Handed-ness	Epileptic Focus	EEG Findings	MRI findings	Medication at day of HD-EEG^a^	Generator	SEEG	Surgery, Outcome > 1 year	Pathology
1	26, F	R	L mesio-temporal	Interictal: T3, T9, FT9, FT7, F9 > T5, F7 (frequent) or F10, T10, T4, F8, Zy2, P10 *Ictal: L posterior temporal (T3, T5, P9)	Normal	Lamotrigine 550 mg Lacosamide 500 mg Perampanel 8 mg	Deep	X	RFTC, Ia	
2	18, F	R	R temporo-occipital	Interictal: PO8 > P12, T6, P10, O2, PO4 *Ictal: R temporo-occipital (T6, P10, O2)	Normal	Carbamazepine 1000 mg Perampanel 8 mg	Intermediate	X	R temporo-occipital resection, n.a	FCD IIa
3	41, F	R	R mesio-temporal	Interictal: F8, T4, F10, T10, FT8, FT10, FT12 (frequent) or F7, T3, F9, T9, FT9, FT7 *Ictal: R temporal (F8, T4, F10, T10) or bitemporal (F8, T4, F10, T10 and F7, T3, F9, T9)	R MTS	Lacosamide 200 mg	Deep		R anterior temporal lobectomy, Ia	Hippocampal sclerosis
4	25, F	L	L mesio-temporal	Interictal: F7, F9, T9, FT9, F11, FT11 *Ictal: L temporal (F7, T3, F9, T9, Zy1)	L MTS	Lamotrigine 100 mg	Deep			
5	51, F	R	R mesio-temporal	Interictal: F8, T4, F10, T10, FT10, AF8, F12 *iEEG: R temporal (F8, T4, F10, T10, Zy2)	R hippocampal atrophy	Levetiracetam 3000 mg	Deep	–	R anterior temporal lobectomy, Ia	Hippocampal sclerosis
6	20, M	R	L dorsolateral prefrontal	Interictal: F3, C3, F5, FC5, FC3, FC1 *Ictal: L fronto-temporal (F3, C3 > F7, T3, F9, T9)	L frontocentral ischemic encephalo-malacic lesion	Lacosamide 400 mg Lamotrigine 175 mg	Superficial	–	L dorsolateral frontal resection, Ia	Gliosis, FCD IIa
7	23, M	R	R parietal operculum/posterior insula	Interictal: C4, P4, Pz, CPz, P2, P6, CP2, CP4, CP6 *Ictal: R centro-parietal (C4, P4, Pz)	R parietal operculum/ posterior insula FCD	Carbamazepine 1600 mg Levetiracetam 1000 mg	Intermediate	–	–	
8	24, M	R	R frontopolar	Interictal: Fp2, SO2, AF8, AF4, AFz, F4, Fz > Fp1, F3, AF7, AF3 *Ictal: R frontopolar (SO2, Fp2)	R frontopolar FCD	Carbamazepine 800 mg	Superficial	–	–	
9	43, F	R	R parietal	Interictal: TP8, FT12, FT10, P10, T4, T6, T10 *Ictal: R hemisphere	Normal	Lacosamide 100 mg BID Levetiracetam1500 mg Carbamazepine 1100 mg	Superficial	X	–	
10	40, F	R	L posterior temporal	Interictal: F7, T3, T5, F9, T9, Zy1 *Ictal: L posterior temporal region (T5, T3, P9)	Normal	Lacosamide 100 mg	Deep	–	–	
11	43, M	L	R mesio-temporal	Interictal: F8, T4, F10, FT8, FT10 *Ictal: R temporal (F10, T10, F8, T4, Zy2)	R MTS	Lamotrigine 75 mg Carbamazepine 400 mg	Deep	–	–	
12	35, M	R	L mesio-temporal	Interictal: F7, F9, FT9, F11, FT11 > Fp1, SO1 *Ictal: L temporal region (F7, T3, Zy1)	L temporal lobe atrophy affecting mesial structures	Clobazam 10 mg	Deep	–	–	
13	43, F	R	R insula	Interictal: spike negative *Ictal: R posterior temporal region (T6, P4, P10)	Severe bilateral frontal atrophy	Oxcarbazepine 2100 mg Clobazam 30 mg	Deep	X	–	
14	51, M	R	L mesio-temporal	Interictal: spike negative *Ictal: L temporal (F9, T9, Zy1 > F7, T3)	L mesio-temporal FCD or low-grade tumor	Lacosamide 400 mg	Deep	–	–	
15	40, F	R	R frontal operculum	Interictal: Fp2, SO2, F10, AF8, Zy2, Fpz *Ictal: no EEG changes^b^	R prefrontal cavernoma	Eslicarbazepine 1200 mg Lamotrigine 350 mg Clobazam 30 mg Primidone 125 mg	Superficial	X	R fronto-operculum resection, Ia	FCD IIa
16	21, F	R	R mesio-temporal	Interictal: spike negative *Ictal: R hemispheric	Normal	Lamotrigine 400 mg Clobazam 10 mg	Deep	X	R anterior temporal lobectomy, n.a	FCD I
17	24, M	Ambi-dextrous	L mesio-temporal	Interictal: spike negative *Ictal: L Temporal (F7, T3, F9, T9)	L mesio-temporal cystic lesion	Levetiracetam 3000 mg Lamotrigine 375 mg Lacosamide 400 mg	Deep	–	–	
18	19, F	R	R temporo-occipital	Interictal: T4, T6, TP8, TP10 > C4, P4 *Ictal: R temporo-occipital (O2 > T6, P10, P4)	R lingual encephalo-malacic lesion	Lacosamide 200 mg Levetiracetam 1000 mg	Deep	–	R occipital resection, n.a	Mild FCD
19	32, M	R	R posterior temporal/ posterior insula	Interictal: T4, T6, P12, PO8, TP8 *Ictal: R posterior temporal (T4, T6, T10, P10)	R temporal neocortex atrophy & atrophy/ agenesis of the right piriform	Lacosamide 250 mg Brivaracetam 100 mg Eslicarbazepine 800 mg	Intermediate	X	–	
20	27, F	L	R latero-occipital	Interictal: P10, T6, O2 > PO8, TP10, TP8, TP12, F8, T4 *Ictal: R posterior temporal (T6, P10)	Normal	Levetiracetam 3250 mg Lacosamide 400 mg	Superficial	X	–	
21	40, M	R	L mesio-temporal	Interictal: FT11, FT9, T9, P9, TP9, P11, TP11, P9, TP9, T9 *Ictal: L temporal (F7, T3, F9, T9)	L fusiform lesion	Levetiracetam 2000 mg Carbamazepine 1200 mg	Deep	X	–	

*F* female, *M* male, *R* right, *L* left, *MTS* mesial temporal sclerosis, *FCD* focal cortical dysplasia, *RFTC* radiofrequency thermocoagulation, *n.a.* not applicable (follow up duration < 1 year).

*Ictal data were recorded during the stay in the monitoring unit, no seizure was registered during the night of the HD-EEG recording.

^a^Antiseizure medication was not significantly different between the time of the HD-EEG recording and the day of neuropsychological testing.

^b^This patient does not have significant EEG changes at the time of the seizures and the focus was found to be superficial. In order to see epileptic activity at the scalp level in the EEG, an area > 8–10 cm^2^ of activation is required even if the generator is superficial^16^. SEEG confirmed at the moment of the clinical manifestation that the ictal activity was very focal and only involved a few electrode contacts.

### HD-EEG overnight recordings

**Figure 1:**
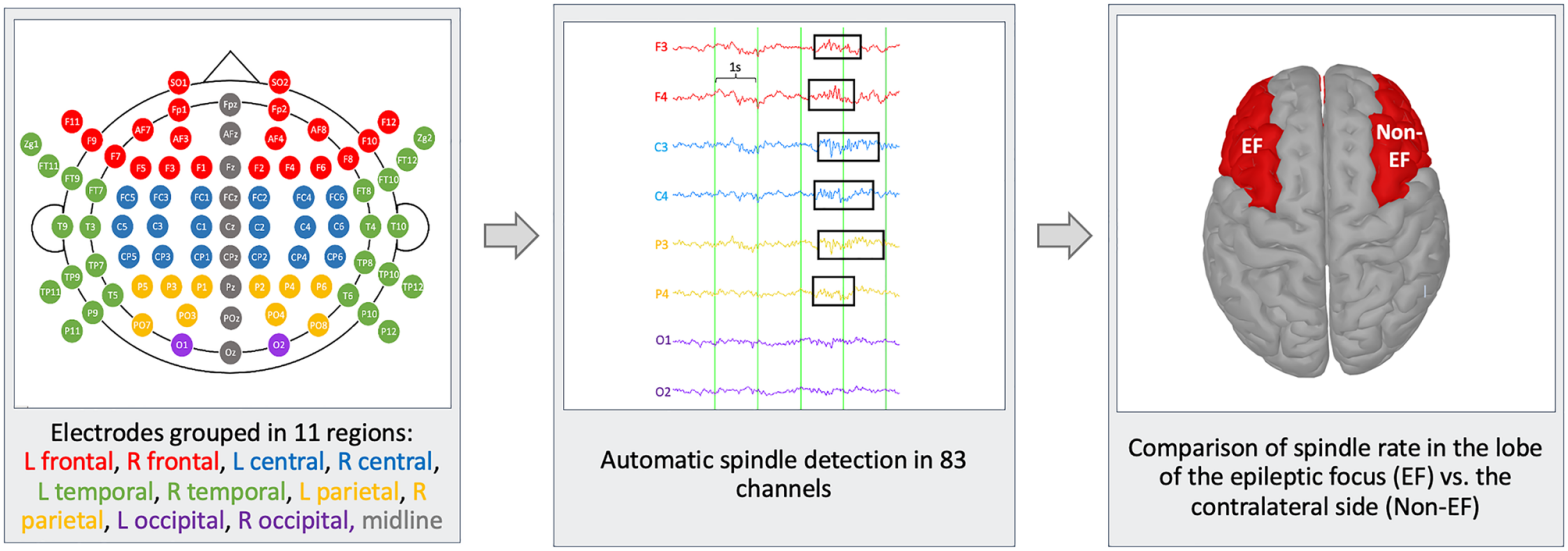
Workflow of the study procedures.

The HD-EEG data of epilepsy patients were obtained in the Epilepsy Monitoring Unit at the Montreal Neurological Hospital. Overnight recordings were performed with the Nihon Koden system (Tokyo, Japan) with 83 glued electrodes placed according to the 10–10 EEG system and a sampling rate of 1000 Hz. Patients were hospitalized for long-term EEG monitoring as part of their presurgical evaluation and HD-EEG was performed on average at day 3 of the hospitalization. Day 1 was chosen only if the patient had already had a previous admission to our unit to counteract a potential first night effect. No seizures occurred during the overnight recordings. HD-EEG data of healthy controls were recorded with a 256-channel EGI system (Electrical Geodesic Inc., EGI, now Magstim EGI, Eden Prairie, MN, USA) at a 1000 Hz sampling rate. The 10–10 system was approximated from the full montage as done in our previous work^17^. Electrodes were grouped into 11 regions: five regions per hemisphere (frontal, central, temporal, parietal, occipital) and midline (see workflow in Fig. 1) to allow a regional comparison of spindle rates.

### Sleep scoring and spike marking

Sleep was manually scored (K.S. and B.F.) in the Stellate Reviewer software (Montreal, Canada) according to AASM criteria^18^ in 30-s epochs displaying EEG using a mastoid reference montage, electrooculography (EOG) and chin electromyography (EMG). We selected the first consecutive 40 30-s epochs of N2 sleep (early N2) and the last consecutive 40 30-s epochs of N2 sleep (late N2) to study the effect of sleep homeostasis on global (whole-brain) and focal (specific brain regions) spindle distribution. Arousals and artifacts were manually marked and excluded from the automatic detection of spindles. Spikes were marked at their peak across all channels by a board-certified neurophysiologist (B.F.) using a bipolar montage.

### Automatic detection of sleep spindles

Spindles were automatically detected with a validated spindle detector^19^ in the combined mastoid reference montage in all 83 channels with the criteria of a frequency of 10–16 Hz and a duration of 0.5–3 s using a custom script written in MATLAB 2020b implemented in our lab. This detector is based on increases of power in the 10–16 Hz band and has been previously used in patients with epilepsy^20^. A smoothing moving window of 300 ms and 75th percentile thresholds was applied. For each participant, detections were visually cross-checked by a board-certified neurophysiologist (B.F.). Spindle rates were calculated per channel and averaged over all channels per region according to the electrode grouping in 11 regions. Further, spindles were categorized as fast spindles (12–16 Hz) or slow spindles (10–12 Hz).

### Neuropsychological assessment

Neuropsychological data were obtained from routine clinical investigations performed by certified neuropsychologists (J.C. and V.S.). We selected tests of memory and processing speed a priori, as we aimed to assess correlations with fast spindles specifically and both tests have been shown to be correlated with fast spindle rates in the literature^7,8,12^. The average time difference between clinical testing and HD-EEG overnight recording was 2.7 ± 3.3 months. In general, neuropsychological measurements are robust over time and are not expected to change over the course of a few months in patients with epilepsy^21,22^ and spindle rates have been shown to be consistent across different nights of a multi-night EEG recording^23^. Verbal memory was assessed with the Nicole Abstract Verbal Learning Test^24^. Subjects had to learn 15 abstract words presented in five learning trials, and an interference list presented once. Following an immediate recall of the interference list, patients were asked to recall the word list. The delayed recall of the word list was performed after 30 min. Results for the verbal memory task are reported as percentage of correctly acquired words in the delayed recall of the word list. Sustained visual attention was assessed with the Ruff 2 and 7 Selective Attention Test with subscales for automatic search and controlled search for speed and accuracy^25^. In this paper–pencil test that measures different components of attentional processing, subjects were asked to cross out the numbers 2 and 7 while ignoring distractors (a categorically different distractor in the “automatic detection” condition or a categorically similar distractor in the “controlled search” condition). Subsequently, speed performance (number of targets detected) and accuracy (percentage of detected targets relative to the total of targets detected and missed) were determined. Neuropsychological results for the attention task are reported in standardized T-values (mean 50 ± 10).

### Statistics

Data were tested for normal distribution with the Shapiro–Wilk test and reported as mean and standard deviation (SD) [mean ± SD] in case of normally distributed data or median and range (median [range]) otherwise. Spindle rates were compared between epilepsy patients and healthy controls on a global and regional level using a t-test and Cohen’s d for effect size. Within epilepsy patients, spindle rates in homologous regions were compared using a Wilcoxon-sum signed-rank test. The effect size was calculated using the matched-pairs rank biserial correlation (RC), a recommended estimate of the effect size for paired non-parametric tests such as the Wilcoxon-sum signed-rank test, and whose values range from − 1 to + 1^26^. Correlations between the number of spikes, neuropsychological parameters (verbal memory, speed performance and accuracy) and spindle rates were assessed using the Spearman correlation coefficient. Due to the exploratory nature of the study, no adjustment for multiple comparisons was performed^27^. Statistical analyses were performed using MATLAB 2020a and R studio; a p-value < 0.05 was considered to indicate statistical significance.

## Results

### Sleep macrostructure

Parameters of sleep macrostructure of patients with epilepsy and healthy controls are presented in Table 2. Further, we added a comparison to a larger healthy control group (n = 100) published in Mitterling et al.^28^.

**Table 2:** Sleep macrostructure in patients with epilepsy (n = 21) compared to healthy controls (n = 12) from the current study and compared to a larger healthy control population (n = 100) from Mitterling et al.^28^.

Sleep parameter	Patients with epilepsy (n = 21)	Healthy controls (n = 12)	Difference patients vs. controls (n = 12)	Healthy controls (n = 100) from Mitterling et al.^28^	Difference patients vs. controls (n = 100)
TIB (min)	493.0 [278.1; 721.0]	537.7 [475.9; 590.9]	p = 0.49	480.0 [420.0; 504.0]	p = 0.27
TST (min)	431.5 [213.0; 660.5]	437.5 [225.0; 563.0]	p = 0.83	403.0 [165.5; 458.0]	p = 0.15
Sleep efficiency (% TIB)	85.7 [66.1; 95.7]	81.3 [46.7; 95.3]	p = 0.30	86.4 [36.9; 98.1]	p = 0.79
Sleep onset latency (min)	19.0 [7.0; 54.4]	8.7 [3.0; 36.0]	**p = 0.04, d = 0.43**	16.1 [2.9; 119.0]	p = 0.20
REM sleep latency (min)	154.2 [65.1; 369.5]	180.6 [65.5; 447.5]	p = 0.11	119.0 [49.5; 396.5]	p = 0.01
N1 (% TIB)	6.4 [2.3; 14.6]	7.7 [2.5; 23.6]	p = 0.78	9.2 [3.3; 34.6]	**p = 0.04, d = -0.27**
N2 (% TIB)	40.0 [13.7; 51.1]	38.3 [24.0; 55.0]	p = 0.99	42.2 [15.5; 58.9]	p = 0.07
N3 (% TIB)	22.6 [11.0; 28.2]	19.9 [9.6; 45.7]	p = 0.24	15.9 [0; 36.6]	**p < 0.001, d = 0.54**
REM (% TIB)	17.7 [4.5; 28.8]	13.0 [0.6; 18.4]	**p = 0.001, d = 0.65**	12.5 [0.9; 24.7]	**p < 0.001, d = 0.55**
WASO (% TIB)	7.2 [1.4; 27.1]	12.9 [2.4; 46.0]	p = 0.32	11.2 [1.8; 58.7]	p = 0.15

*TIB* time in bed, *TST* total sleep time, *WASO* wake after sleep onset.

Significant values are in bold.

### Spindle rates

**Figure 2:**
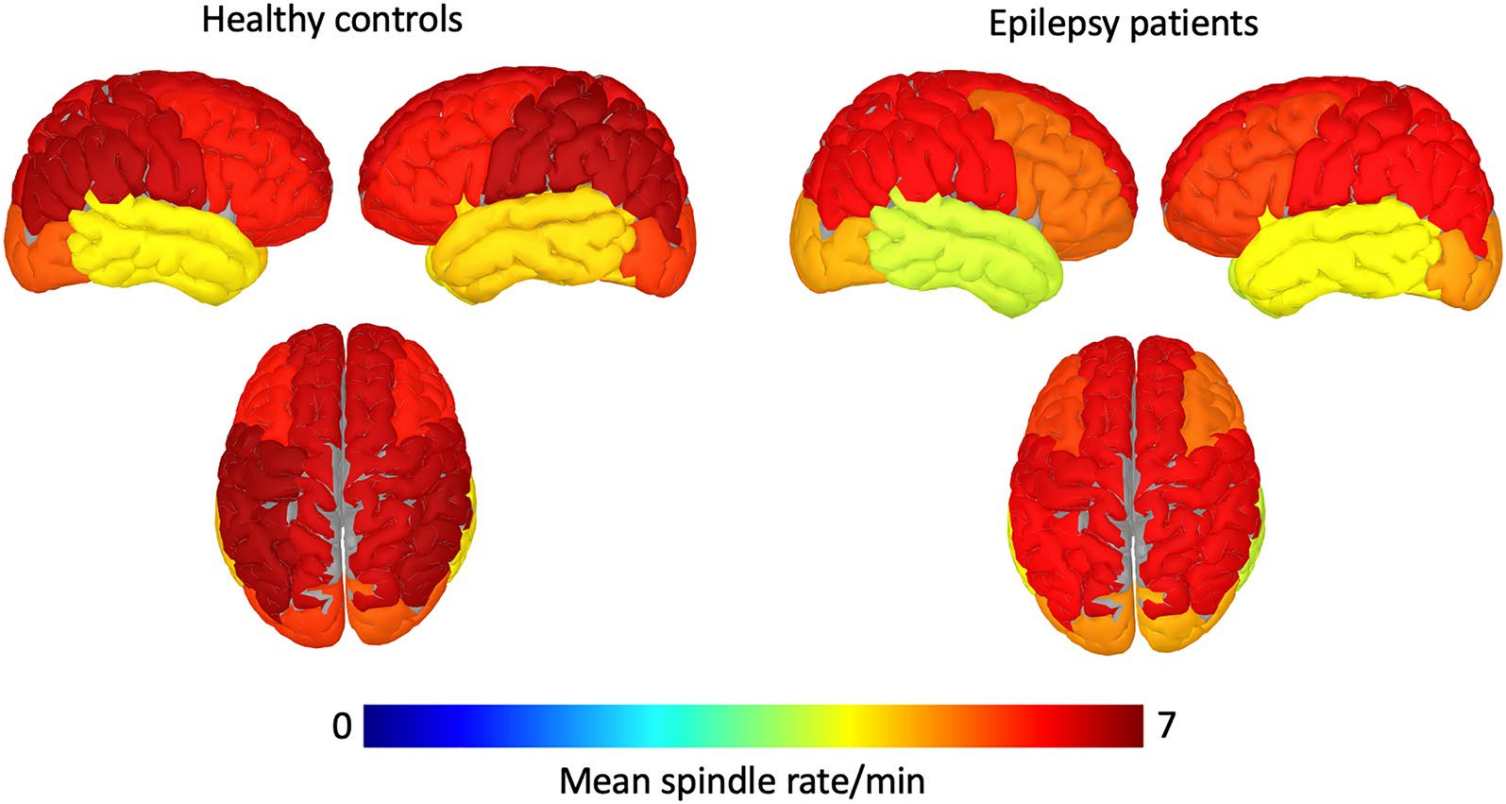
Mean spindle rates per region (frontal, central, temporal, parietal, occipital and midline) in healthy controls and patients with epilepsy for all N2 epochs.

Global spindle rates during N2 sleep were reduced in epilepsy patients compared to healthy controls (5.78/min ± 0.72 vs. 6.49/min ± 0.71, p = 0.02, d = − 0.70). Figure 2 shows that this reduction in spindles was present for 10/11 investigated regions (all p-values < 0.05) and was borderline significant for the midline (p = 0.05). Extratemporal lobe epilepsy patients showed a reduction of global spindle rates in N2 compared to controls (5.57/min ± 0.67 vs. 6.49/min ± 0.71, p = 0.004, d = − 0.93) whereas temporal lobe epilepsy patients were not significantly different than controls (5.99/min ± 0.78 vs. 6.49/min ± 0.71, p = 0.07, d = − 0.47). The maximum rates of slow spindles were over frontal regions, whereas the maximum of fast spindles was observed over central regions.

For patients with epilepsy, spindle rates did not differ significantly between the hemisphere with the epileptic focus and the contralateral hemisphere (5.48/min [4.53–7.03] vs. 5.62/min [4.46–6.94], p = 0.13). On a regional level, spindle rates were lower in the region with the epileptic focus compared to the homologous contralateral region (4.77/min [2.53–6.18] vs. 5.26/min [2.53–6.56], p = 0.02, RC =  − 0.57). This reduction in spindles rates was driven by fast spindles (12–16 Hz) specifically (1.50/min [0.62–4.08] vs. 1.65/min [0.51–4.28], p = 0.002, RC =  − 0.76). There was no difference in slow spindle rates between homologous regions (2.81/min [0.79–5.05] vs. 2.98/min [1.09–4.63], p = 0.52).

### Sleep homeostatic properties of spindles: early versus late N2 sleep

Global spindle rates were significantly reduced in early N2 sleep in epilepsy patients compared to healthy controls (5.45/min ± 0.66 vs. 6.17/min ± 0.75, p = 0.01, d =  − 0.72). These reductions were present in all regions evaluated, reaching statistical significance in seven out of 11 regions (each p < 0.05). In late N2 sleep, epilepsy patients showed no significant reduction in spindle rates compared to healthy controls (6.27/min ± 0.80 vs. 6.81/min ± 0.82, p = 0.06).

**Figure 3:**
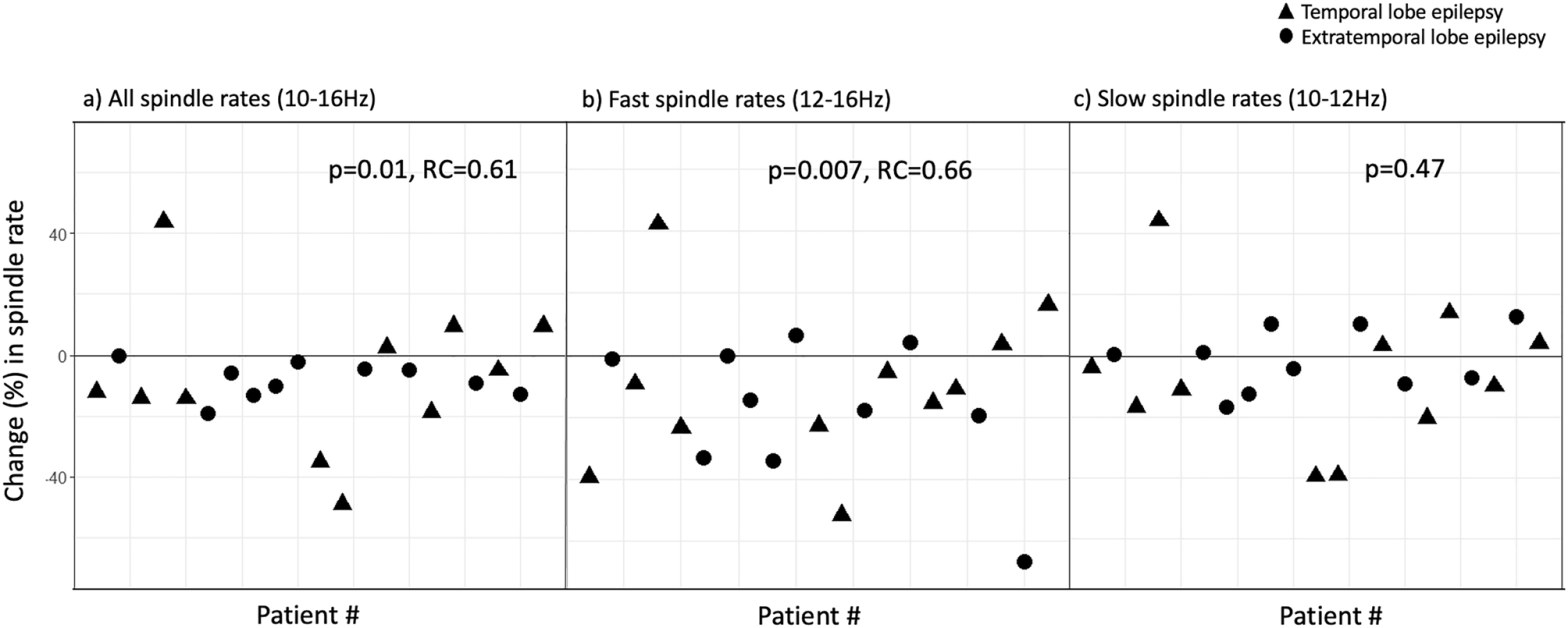
Decrease (%) in (**a**) all spindle (10–16 Hz), (**b**) fast spindle (12–16 Hz) and (**c**) slow spindle (10–12 Hz) rates for the region with the epileptic focus compared to the contralateral side in early N2 sleep. Each point (circle or triangle) represents a patient.

For epileptic patients, there was a significantly lower spindle rate on a regional level when comparing the region containing the epileptic focus to the homologous contralateral side in early and late N2 with a stronger effect in early N2 sleep (early N2: 4.60/min [1.92–6.15] vs. 5.24/min [1.52–6.77], p = 0.01, RC =  − 0.61; late N2: 4.84/min [2.77–7.77] vs. 5.12/min [3.52–7.36], p = 0.04, RC =  − 0.48). Fast spindles were the driver for this reduction, with a robust effect over both early and late N2 sleep (early N2: 1.46/min [0.45–3.98] vs. 1.56/min [0.35–3.99], p = 0.007, RC =  − 0.66; late N2: 1.95/min [0.55–4.25] vs. 1.77/min [0.63–5.6], p = 0.007, RC = -0.66; *see* Fig. 3). There were no regional differences for slow spindles (early N2: 3.10/min [0.56–4.75] vs. 2.95/min [0.78–4.55], p = 0.47; late N2: 2.88/min [1.03–5.34] vs. 2.97/min [1.40–4.82], p = 0.68).

### Temporal vs. extratemporal lobe epilepsy

To study the influence of the location of the focus, analyses were performed for temporal and extratemporal lobe epilepsy patients separately. For the 11 temporal lobe epilepsy patients, spindle rates in total N2 sleep were reduced in the region with the epileptic focus compared to the contralateral side (5.21/min [4.40–6.14] vs. 5.65/min [4.36–6.56], p = 0.003, RC =  − 0.57). This decrease was also found for fast spindles (1.60/min [0.67–4.08] vs. 1.82/min [0.82–4.14], p = 0.03, RC =  − 0.98). The effect of the reduction in the region with the epileptic focus compared to the contralateral side was stronger for spindles in early N2 compared to late N2 (early N2: 4.68/min [4.06–6.15] vs. 5.30/min [4.35–6.77], p = 0.002, RC =  − 0.99; late N2: 5.71/min [4.63–6.21] vs. 5.98/min [4.35–6.89], p = 0.03, RC =  − 0.74).

For the 10 extratemporal lobe epilepsy patients, there were no significant differences in spindle rates between the region with the epileptic focus and the homologous contralateral side in total N2 sleep (4.20/min [2.35–6.18] vs. 4.95/min [2.52–5.92], p = 0.24), early N2 sleep (4.59/min [1.92–5.87] vs. 4.47/min [1.53–5.76], p = 0.30), and late N2 sleep (4.35/min [2.77–7.77] vs. 4.47/min [3.52–7.36], p = 0.27). There was a significant reduction in fast spindle rates in the region with the epileptic zone compared to the contralateral homologous region in total N2 sleep (1.41/min [0.62–2.91] vs. 1.81/min [0.82–4.14], p = 0.01, RC =  − 0.74).

### Spike rates and correlation with spindle rates

Four of the 21 epilepsy patients (19%) had no spikes present in their scalp HD-EEG data. Spike rate was significantly higher in early compared to late N2 sleep (3.25/min [0–70.2/min] vs. 2.8/min [0–58.4/min], p = 0.03, RC = − 0.49). Spike rate was not significantly correlated with spindle rate across all N2 epochs (p = 0.77) nor in early (p = 0.53) and late (p = 0.30) N2 sleep. Eight of the 21 patients (38%) had a superficial epileptic focus where one would expect that spikes would be detected in the scalp EEG, an assumption which is not necessarily true for more deep-seated foci. Within these patients with superficial foci, spike rates were neither correlated with spindle rates in all N2 epochs (p = 0.11) nor with the percentage decrease in spindle rates between the region with the epileptic focus compared to the homologous contralateral side (p = 0.89).

### Neuropsychological performance

**Figure 4:**
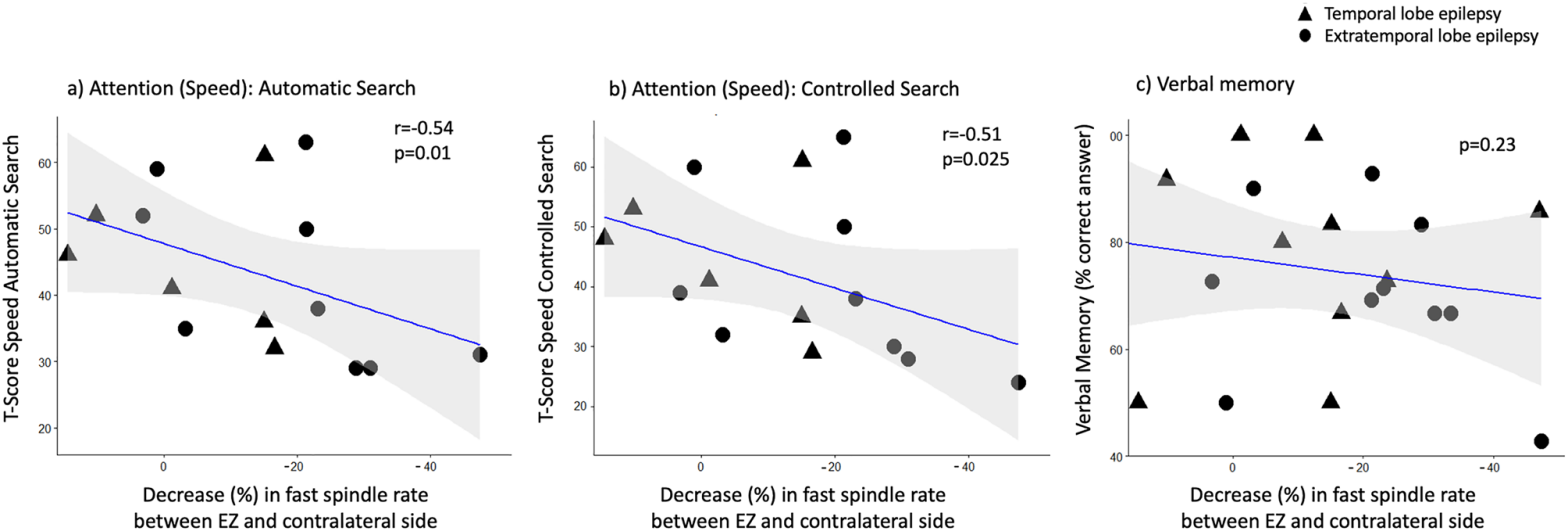
Correlation between decrease (%) in fast spindle rates (region with the epileptic focus compared to contralateral side) in N2 sleep and (**a**) selective attention task for speed subscale “automatic search”, (**b**) selective attention task for speed subscale “controlled search” and (**c**) verbal memory test. Grey shading represents 95% confidence interval.

Neuropsychological findings are reported in Table 3. Verbal memory scores were available for all 21 patients. Attention measures (speed and accuracy) were available for 15 patients. For selective attention, there was a negative correlation between both subscales of speed performance (automatic detection: r =  − 0.54, p = 0.01 and controlled search: r =  − 0.51, p = 0.025) and the percentage decrease in fast spindle rate between the lobe with the epileptic focus and the contralateral side in all N2 epochs (see Fig. 4). No correlations were found for the subscales of accuracy (automatic detection: p = 0.38, controlled search: p = 0.38). Group analysis revealed no significant correlation between verbal memory scores and decreased spindle rates (p = 0.23). Within the subgroup of patients with dominant temporal lobe epilepsy (n = 6), a significant negative correlation was found between memory performance and the percentage decrease in fast spindle rates in N2 sleep (r =  − 0.93, p = 0.004).

**Table 3:** Neuropsychological findings in patients with epilepsy (n = 21).

ID	Visual attention—speed (automatic detection)	Visual attention—speed (controlled search)	Visual attention—accuracy (automatic detection)	Visual attention—accuracy (controlled search)	Verbal memory
1	29	28	55	60	66.7
2	36	35	25	36	50
3	50	50	57	59	92.8
4	n.a.	n.a.	n.a.	n.a.	85.7
5	35	32	51	33	90
6	n.a.	n.a.	n.a.	n.a.	85.7
7	41	41	45	51	100
8	61	61	20	20	83.3
9	32	29	40	38	66.7
10	46	48	51	52	50
11	63	65	45	53	69.2
12	31	24	57	45	42.9
13	n.a.	n.a.	n.a.	n.a.	100
14	29	30	49	55	83.3
15	52	53	54	54	91.7
16	38	38	53	52	71.4
17	n.a.	n.a.	n.a.	n.a.	66.7
18	n.a.	n.a.	n.a.	n.a.	80
19	59	60	52	56	50
20	n.a.	n.a.	n.a	n.a	72.7
21	52	39	53	57	72.7

Performance of visual attention task is reported in T-values, verbal memory performance in % of acquired words retained at delayed recall.

*n.a.* not available.

## Discussion

This study provides insights into the global and local disturbances of spindles, the influence of sleep homeostasis, and the functional correlates of such disturbances in patients with various types of focal epilepsies. Using combined polysomnography and HD-EEG, we investigated global and local changes in rates of spindles in relation to the anatomical location of the epileptic focus and correlated these findings with neurocognitive performance. The most relevant findings of the current study are that (i) there is a reduction in spindle rates in epilepsy patients compared to healthy controls and a local decrease in spindle rates in the region with the epileptic focus compared to the contralateral side, (ii), there is a sleep homeostatic effect on spindle and spike rates across the night, and (iii) there is a negative correlation between a local reduction in spindles and neurocognitive performance measures.

### Overall spindle rate and distribution are impaired in focal epilepsy

Our work is the first to show a reduction of spindles in patients with various types of drug-resistant focal epilepsy on a global and regional level compared to healthy controls and additionally at the site of the epileptic focus as compared to the contralateral homologous brain region. There are few studies in various epilepsy types using different methodology with only two studies having a healthy control group^9–12,29^. The evidence from these studies suggests that epilepsy impacts the presence of spindles, with most knowledge obtained in idiopathic generalized epilepsy and specific childhood epilepsy syndromes. A global reduction in spindle rates has been previously reported in a pediatric epilepsy population relative to a healthy control group^10^. Our work revealed a global reduction in spindle rates for adult patients with focal epilepsy compared to healthy controls over all N2 epochs with significant spindle reductions in both early and late N2 sleep. Although sleep macrostructure may have an influence on spindle distributions, this did not differ between epilepsy patients and controls. An exception to this was in the case of sleep latency, the amount of REM sleep in comparison to our healthy control group and the amount of N1, N3 and REM sleep in comparison to a larger healthy control population^28^. However, sleep latency was not reduced in epilepsy patients in comparison to the larger healthy control group of Mitterling et al.^28^ and the younger age of our study population in comparison to Mitterling et al.^28^ may explain the higher amount of N3 sleep. Overall, the spindle decrease is unlikely to be confounded by parameters of sleep macrostructure. Furthermore, it would not explain the focal reduction in spindles which was found at the site of the epileptic focus.

Within epilepsy patients, spindle rates were reduced in the region containing the epileptic focus. These findings, despite being generated in our heterogeneous group of focal epilepsy patients on variable medications, add new knowledge to the previous findings in children with childhood epilepsy with centrotemporal spikes^12^. Further, the local reduction in spindle rates was particularly pronounced for fast spindles. Interestingly, fast spindles play an important role in cognition^7^ and are reduced in other neurocognitive diseases such as Alzheimer’s disease^30^ and schizophrenia^8^. Although exploratory, our findings support growing evidence suggesting that local brain pathology, such as that which may be present in epilepsy, is not only associated with an increase in pathological markers of disease such as epileptic spikes or pathological slow waves, but also with a reduction of physiological activity such as spindles. This latter hypothesis is in keeping with an intracranial EEG study which showed that there is an inverse relationship between epileptic spikes and spindles in the ipsilateral hippocampi of patients with temporal lobe epilepsy^31^.

### Fast spindle reduction is correlated with cognitive performance

Local reductions in fast spindles were correlated with lower cognitive performance such as speed performance. This is the first study in a group of heterogenous focal epilepsy patients showing a correlation between spindle decreases and neurocognitive function. Our results are consistent with a previously demonstrated relationship between fast spindle rates and neuropsychological performance^7,8^ and with findings of negative correlations between spindle rates in the area of the epileptic focus and intelligence quotient and executive functions in childhood epilepsy with centrotemporal spikes^12^. In the current study, focal epilepsy patients with more pronounced spindle reductions showed greater decreases in speed performance, whereas there was no effect on accuracy. Patients showed this reduction in both subscales measuring speed (controlled search and automatic detection). This points to the fact that the spindle decrease might have a more general effect on psychomotor processing speed. Spindles have their maximum over the fronto-centro-parietal regions^32^ and around 50% of spindle events are symmetrically distributed over both hemispheres^23^. Given that fast spindles are associated with processing speed^8^, a reduction of spindles in the brain region with the epileptic focus may therefore have an impact on processing speed due to the imbalance of spindle distribution. On the verbal memory task, only the subgroup of patients with left temporal lobe epilepsy showed a negative correlation between spindle reductions and memory performance. The relationship between impairment of verbal memory in temporal lobe epilepsies is well known^33^. Nevertheless, the concurrent spindle reductions and decreased neuropsychological parameters found in our study does not allow the presumption of a causal link between those two factors.

### Temporal and extratemporal lobe epilepsies have different spindle reduction profiles

Patients with extratemporal lobe epilepsy had significant decreases in all spindle and fast spindle rates in the region with the epileptic focus. Fronto-centro-parietal brain areas are known to have maximal rates of spindles compared to other brain regions^32^, thus it is possible that electrophysiological investigations in extratemporal epilepsies are better able to capture reductions in spindle rates. In contrast, patients with temporal lobe epilepsy showed only a decrease in fast spindle rates in the region with the epileptic focus.

### Sleep homeostatic properties of spindles and spike rates

It is well known that homeostatic processes are implicated in sleep regulation^34^. In our study, there was a homeostatic spindle profile, with more regional reduction of spindles in early compared to late N2 sleep. Interestingly, the decrease in fast spindles in patients with epilepsy was sustained throughout the night. Due to the correlation of fast spindles with cognitive performance^7^, this reduction in spindles may underlie, at least in part, the cognitive comorbidities frequently encountered in patients with epilepsy^35^.

Spike rates were higher in the beginning of the night compared to the end of the night in patients with epilepsy. This points to the fact that spike rates are under the control of sleep homeostasis processes. In children with electrical status epilepticus in sleep, the overnight decrease in sleep slow waves is higher outside the epileptic focus compared to the decrease in the area of the epileptic focus^36^. Therefore, epileptic activity seems to influence the homeostasis of physiological sleep oscillations such as sleep slow waves depending on the location of the epileptic focus. Conversely, spikes are modulated by sleep slow waves, and we recently showed that spikes and high-frequency oscillations, used as novel markers of the epileptogenic zone, were strongly correlated with higher amplitude slow waves^4^. Given that slow wave power is higher in early compared to late sleep^37^, a higher spike rate in early sleep can be well explained. To the best of our knowledge, the only evidence pointing to an overnight decrease in spikes is from children with epilepsy showing lower centrotemporal spike rates towards the end of the night^38^. A potential explanation for the more pronounced decrease in regional spindles in early versus late N2 sleep could be a higher rate of spikes in the beginning of the night. This was, however, not confirmed in the present study, as we found no correlation between the spike rate and the regional reduction in spindles. This is potentially explained by the underlying neuropathology in our sample of patients with epilepsy^39^ which may have caused the disruption in spindle generation and cognitive function.

### Reduction of sleep physiology as a potential marker of the epileptogenic zone

Until now, biomarkers used to define epileptogenicity are based on pathology such as the presence of epileptic spikes, high-frequency oscillations as novel biomarkers of the epileptogenic zone, or pathological slow waves^40^. Our work calls for a paradigm shift whereby clinicians should consider not only the presence of pathological markers but the absence of physiological markers to aid in the identification of the seizure focus in patients with epilepsy. Based on our findings, the local reduction of sleep spindles could be further explored not only as a marker of the epileptic focus but also as a follow-up marker after medical or surgical intervention, and postsurgical persistence of local reductions may be predictive of seizure relapse.

One main limitation of the study is that our study does not confirm whether spindles or an underlying third factor are the primary driver of cognitive dysfunction in patients with epilepsy. As known from intracranial EEG studies, not all spikes, particularly those from deep foci, can be recorded from the scalp^15^. Furthermore, while spindle rate is a stable intra-individual characteristic, variations in spike rate can occur from night to night^41^. This might also potentially be a reason for not finding a correlation between spike and spindle rates. Additionally, spindle analyses were performed solely in N2 sleep. This was decided based on the fact that spindles are more frequent in N2 sleep than in N3 sleep^23^. Further, the results were not corrected for multiple comparisons due to the exploratory nature of our study. The number of participants with available data for attention performance was low with no possibility to compare the neuropsychological performance to a healthy control group. Our findings shall be therefore strengthened in future studies with a larger sample size and neuropsychological testing in a control group. Finally, a previous study found that spindle rates were 0.16 times higher in women^23^. There was a higher percentage of male subjects in our control group (75%) compared to the epilepsy group (43%), so it is possible that differences in spindle rates between these groups may have been underestimated due to the gender characteristics of our study’s samples.

## Conclusion

Spindle structure and function are impaired in patients with focal epilepsy at both the global level as well as the region of the epileptic focus with particularly reduced fast spindle rates. This decrease in spindle rates correlates with neurocognitive performance and might be one contributing factors to neurocognitive dysfunction which is frequently encountered in people with epilepsy. Future studies may seek to assess local spindle decreases in patients with epilepsy and use these reductions as a potential preoperative marker of the epileptic focus and postoperative marker (or lack thereof) for surgical success.

## Data Availability

The datasets generated during and/or analyzed during the current study are available from the corresponding author on reasonable request.
